# Use of black carp (*Mylopharyngodon piceus*) in biological control of intermediate host snails of fish-borne zoonotic trematodes in nursery ponds in the Red River Delta, Vietnam

**DOI:** 10.1186/1756-3305-6-142

**Published:** 2013-05-16

**Authors:** Nguyen M Hung, Nguyen V Duc, Jay R Stauffer, Henry Madsen

**Affiliations:** 1 Department of Parasitology, Institute of Ecology and Biological Resources, Vietnam Academy of Science and Technology, 18 Hoang Quoc Viet, Hanoi, Vietnam; 2 Ecosystem Science and Management, Pennsylvania State University, 432 Forest Resources Building, University Park, PA 16802-4300, USA; 3 Centre for Health Research and Development, Department of Veterinary Disease Biology, Faculty of Health and Medical Sciences, University of Copenhagen, Thorvaldsensvej 57, Frederiksberg C 1871, Denmark

**Keywords:** Molluscivore fish, *Clonorchis sinensis*, Intestinal trematodes, Transmission, Aquaculture

## Abstract

**Background:**

The risks of fish-borne zoonotic trematodes (FZT) to human health constitute an important problem in Vietnam. The infection of humans with these trematodes, such as small liver trematodes (*Clonorchis sinensis* and *Opisthorchis viverrini*), intestinal trematodes (Heterophyidae) and others is often thought to be linked to fish culture in areas where the habit of eating raw fish is common. Juvenile fish produced in nurseries are often heavily infected with FZT and since fishes are sold to aquaculture facilities for growth, control of FZT in these fishes should be given priority. Controlling the first intermediate host (i.e., freshwater gastropods), would be an attractive approach, if feasible. The black carp, *Mylopharyngodon piceus*, is a well-known predator of freshwater snails and is already used successfully for biological control of snails in various parts of the world including Vietnam. Here we report the first trials using it for biological control of intermediate host snails in nursery ponds stocked with 1-week old fry (10–12 mm in length) of Indian carp, *Labeo rohita*.

**Methods:**

Semi-field and field experiments were set up to test the effect of black carp on snail populations. In the semi-field experiment a known quantity of snails was initially introduced into a pond which was subsequently stocked with black carp. In the field trial in nursery ponds, density of snails was estimated prior to a nursing cycle and at the end of the cycle (after 9 weeks).

**Results:**

The results showed that black carp affect the density of snail populations in both semi-field and field conditions. The standing crop of snails in nursery ponds, however, was too high for 2 specimens to greatly reduce snail density within the relatively short nursing cycle.

**Conclusions:**

We conclude that the black carp can be used in nursery ponds in Northern Vietnam for snail control. Juvenile black carp weighing 100 - 200g should be used because this size primarily prey on intermediate hosts of FZT and other studies have shown that it does not prey on fish fry of other species. It may be necessary to use a high stocking density of black carp or to reduce snail density in the nursery ponds using other measures (e.g. mud removal) prior to stocking fry in order for the black carp to keep the density of intermediate host snails at a very low level.

## Background

Liver and intestinal trematodes are important public health concerns in many Asian countries [1-3]. People become infected when they eat raw or insufficiently cooked fish containing the infective stage, metacercariae [4-6]. The pathology caused by liver trematode infections in people can be quite severe [2], while infections with intestinal trematodes generally are considered to be less severe [7].

Vietnam is endemic for small liver trematodes, *Clonorchis sinensis* (Cobbold, 1875) and *Opisthorchis viverrini* (Poirier, 1886), with an estimated 2 million people infected [8,9]. While there is no estimation of the number of people infected with intestinal trematodes, such as *Haplorchis pumilio* (Looss, 1886), *Centrocestus formosanus* Nishigori, 1924 and others, the prevalence is believed to be high, e.g. in the Red River Delta and some provinces in central Vietnam [6,10,11].

Light infections by *C*. *sinensis*, *O*. *viverrini* or *O*. *felineus* (Rivolta, 1884) are asymptomatic, but high levels of infection and chronic infection cause damage to the bile duct epithelium, eliciting gastrointestinal problems, damage to the liver and possibly cholangiocarcinoma [2,12-14]. *Opisthorchis viverrini* and *C*. *sinensis* have been rated as class 1 carcinogens by the International Agency for Research on Cancer [15]. Lun *et al*. [16] estimated that 35 million people globally could be infected by *C*. *sinensis*, while more than 11 million people are infected with *Opisthorchis* spp. [17]. Epidemiology and pathogenesis of intestinal trematodes are not well understood. Compared to liver trematodes, infection with intestinal trematodes does not generally present significant clinical symptoms [7,12], however, some Heterophyidae species can cause significant pathology, often fatal, in the heart, brain and spinal cord of humans [18]. It is thought that approximately 18 million people could be infected globally by these species [5].

The trematodes not only cause pathology in the final hosts, they also adversely impact on the intermediate hosts. Trematode infection in snails reduces survival and reproduction [19,20]. Large numbers of *Haplorchis pumilio* cercariae can be lethal to fry of *Oreochromis niloticus* (Linnaeus, 1758) within a few hours [21] and high loads of *Centrocestus formosanus* metacercariae cause morbidity in their fish hosts [22], reducing their growth and increasing mortality. Presence of metacercariae in fish raised in aquaculture not only pose a health risk for people eating insufficiently cooked or raw fish, but it also reduce marketability of fish [23].

Aquaculture in northern Vietnam is to a large extent dependent on family-based integrated pond systems, the so-called VAC system where a garden (Vuon), a fish pond (Ao) and an animal shed (Chuong) constitute a functional unit. The VAC system is considered as an ecological way of using farm products in a natural cycle [24], which has been encouraged by the Vietnamese government and has had remarkable results from economic, health and nutrition perspective [25]. In this system, manure from the husbandry is used to fertilize ponds, so as to stimulate algal growth and subsequently fish growth, and remnants from garden products and fish remains can be fed to pigs/cattle. Mud from the ponds is used to fertilize gardens or fields [26]. In fish ponds, the main cultured fish are various carp species. Fish for seeding are produced in hatcheries and after about one week, fry is transferred to nursing ponds, where they will be kept for about 9 weeks before being sold for grow-out farmers. Previous studies have shown that fry leaving the hatcheries are free from infection with FZT [27], while transmission is intense in nursing ponds [6,28] and grow-out ponds [6,10,28,29].

Controlling the intermediate host snails should be part of an integrated strategy for control of these infections in fishes. Since nursery ponds are very important for the transmission it would be important to control infections in these ponds. Biological control of snails using molluscivorous fishes, such as the black carp, *Mylopharyngodon piceus* (Richardson, 1846), might be a viable option.

Black carp is a large cyprinid fish, with a maximum reported size of about 1.5 m and a weight of 70 kg [30]. The native range of black carp includes most major Pacific Ocean drainages of eastern Asia from the Amur River Basin to the West-Pearl River Basis and the Red River of northern Vietnam. Larvae and small juveniles feed almost entirely on zooplankton and aquatic insects while larger juveniles and adults feed mainly on molluscs [30]. The shift in diet occurs with the development of pharyngeal teeth at lengths from 3–33 cm, depending on the development rate of the fish and abundance of prey available [30]. The black carp is already used for biological control of snails in various parts of the World [30-35]. The major concern about using black carp outside its natural distribution is its potential spread to large rivers, where it may establish populations, with potential severe effect on native mollusc populations [30].

We attempted to use black carp in nursery ponds yet this could present a major challenge because the black carp used, should have an appreciable size compared to the fry, so that the fry are protected as the larger carp tend to consume the intermediate host snails (species of Thiaridae and Bithynidae). One concern about using black carp in nursery ponds is that it might switch diet from snails to the fry that they are supposed to protect from infection. Thus it has been observed that fish, which in their natural habitat are specialized molluscivores, will switch to a softer diet when introduced into ponds for biological control [36]. Hung [37] showed that juvenile black carp could be used in nursery ponds without affecting survival of fry or juvenile fishes. Disturbances to the pond environment caused by the black carp, however, might affect fry survival; for example suspension of bottom sediment in the water due to its feeding at the bottom. This paper describes two field trials using black carp to control intermediate host snails in (1) a pond without other fishes and (2) in real nursery ponds.

## Methods

### Semi-field experiment

An experimental pond (20m wide × 30m long × 1.6m deep) at The Research Institute for Aquaculture no. 1 (RIA 1), Tu Son, Bac Ninh was used in this trial from May to August, 2011. The pond was divided into 2 equal parts 15 × 20m by a net with a mesh size of 2 × 2cm across the pond. The mesh size allowed snails to pass from one area to the other, while black carp could not.

Five exclosures (2 × 1 × 1.7m) with a mesh size of 2 × 2cm were established in each part of the pond. The exclosures were fixed to a bamboo pole in each corner and in each corner of the exclosure a brick was placed to ensure that the bottom part stayed covered by a 2-3cm layer of bottom sediment. The water level in the pond was kept at 1.2m. A total of 12,763 thiarid snails - primarily *Melanoides tuberculata* (Müller, 1774) and 6,479 viviparid snails - primarily *Angulyagra polyzonata* (Frauenfeld, 1862) were introduced into the pond over some days. The total weight of snails released evenly over the entire pond was 25kg. Thereafter, 5 specimens of black carp (700 - 1500g) were released in one part of the pond and 10 specimens in the other part. After three months, the pond was emptied. Mud inside each exclosure was removed to a depth of 5cm and sieved to retain snails, which were then counted. Next to each exclosure, mud was similarly removed and sieved from an area of the same size as the exclosure, i.e. 2 m^2^. All live snails were collected and counted. After that, the entire bottom of the pond was checked again to find any live snails. The black carp specimens were anesthetized before euthanization. All specimens were preserved in formalin and stomachs were later examined for content.

### Field trial in real nursery ponds

Three households, which had two similar nursery ponds (size, depth and dense snail population), were selected in Hai Quang commune, Hai Hau district, in Nam Dinh Province. One pond in each household was selected (by flipping a coin) for stocking with black carp and the other would serve as its control. The surface area of the ponds ranged from 200 (20 × 10m) to 360 square meters (20 × 18m). Water was pumped into the ponds from a small canal (1–1.5 m wide) close to the pond. There was a crude filter preventing coarse material entering the pump but there was no filtering at the inlet tube to the pond, therefore some snails could have entered the pond during the process. Many farmers, however, use a filter on the inlet pipe [38], but the mesh size is not fine enough to prevent juvenile snails from entering the pond.

After filling, snail population density was estimated immediately before starting the experiment and again at termination, using a dredge 0.25m wide mounted on a 3m long handle [38]. The dredge was dragged from 2m off shore perpendicularly back to the pond bank. Thus, the area sampled was 0.25 × 2m with a total of 6 such samples obtained from each pond. Each sample was washed and all live snails were counted and identified to species using keys by Thanh [39] and Brandt [40].

The experiment started in June, 2011 when 30,000 fry of Indian carp, *Labeo rohita* (Hamilton, 1882), were released in each of the 6 ponds. Two specimens of black carp (weight: 400 - 1100g) were released in the selected intervention pond in each household. After 9 weeks (the normal duration of the nursing period), the experiment was terminated and all Indian carp fingerlings were caught by seining and transported to another pond. Ponds were then emptied for water.

The density estimated by the dredge can probably only be considered as semi-quantitative and we therefore decided to evaluate this sampling method by quantitative sampling immediately after emptying the pond. The density of snails in the emptied ponds was measured by using an aluminium frame (0.5 × 0.5m) to sample the following distances from the pond bank along a transect perpendicular to the bank, 0 - 50cm, 50 - 100cm, 100 - 150cm and 150 - 200cm. Mud was removed within the demarcated area to a depth of about 5cm and sieved through a mesh 1 × 1mm. These transects did not overlap those that were sampled using the dredge. All snails were counted.

The black carp recaptured were anesthetized, euthanized and preserved in 10% formalin for later examination of stomach content.

### Statistical analyses

Counts of thiarid and viviparid snails were compared between treatments using negative binomial regression [41] specified in a generalized linear model with a log-link function. The ancillary parameter was estimated by full maximum likelihood estimation [41]. The effect of farm, depth, treatment and their interaction were included in the model as an explanatory variable. Differences with p-values below 0.05 were considered significant.

### Ethical considerations

The experimental design was approved by the project review board at Institute of Ecology and Biological Resources, Vietnamese Academy of Science and Technology in April 2011. Fish were handled in accordance with the EU Directive 2010/63/EU for animal experiments http://eur-lex.europa.eu/LexUriServ/LexUriServ.do?uri=OJ:L:2010:276:0033:0079:EN:PDF. Farmers were promised financial compensation if the black carp against expectations significantly affected survival of fry during the nursing cycle.

## Results

### Semi-field experiment

No live snails were found in the part of the pond with 10 black carp specimens, while in the other part with 5 specimens, 20 snails (8 specimens of Thiaridae and 12 specimens of Viviparidae) were found inside the exclosures and only 1 specimen of *A*. *polyzonata* was found outside exclosures. These numbers of snails obviously are too low for statistical analysis. The entire pond bottom was scattered with shell fragments and broken shells.

Stomachs of these black carp specimens were all empty, which was to be expected as the population of snails had been almost eliminated.

### Field trial in nursery ponds

The pre-intervention snail sampling from the 6 ponds combined revealed a total of 520 specimens of the Thiaridae, including 497 specimens of *M*. *tuberculata* plus 23 specimens of *Thiara scabra* (Müller, 1774) and 971 specimens of the Viviparidae, including 925 individuals of *A*. *polyzonata* and 46 specimens of *S*. *aeruginosa* (Reeve, 1863). In addition, 21 specimens of *Pomacea canaliculata* (Lamarck, 1819) (Ampullaridae) were found. Bithynidae (e.g. *Bithynia fuchsiana* (Moellendorff, 1888) and *Parafossarulus manchouricus* (Benson, 1842) were not found.

**Figure 1:**
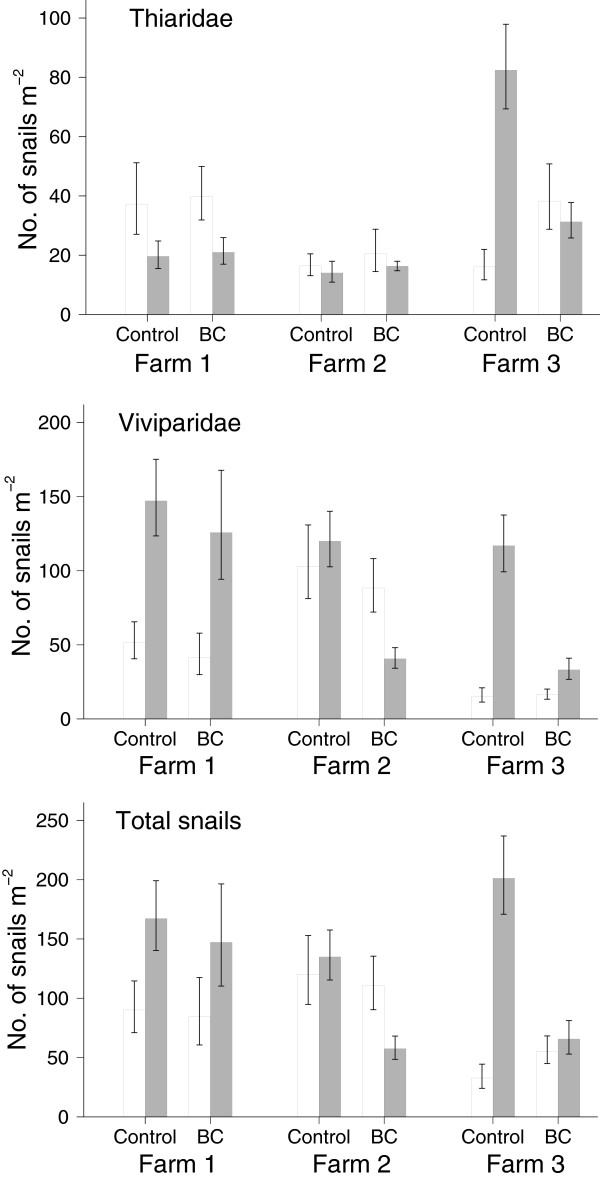
**Snail density in nursery ponds before (open column) stocking with fry and with 2 black carp specimens (BC) or only fry (control) and 3 months later (shaded column) in three households in Nam Dinh Province, Vietnam 2011.** Error bars show 95% confidence limits.

At pre-intervention, counts of thiarid snails (Figure 1) varied significantly among farms (p <0.001), but densities between the two ponds within farms did not differ except in farm 3 where density of thiarid snails in the pond used for black carp was 2.24 times that in the control pond. Viviparid counts differed significantly among farms (p <0.001) but did not differ between control and intervention ponds in any of the three farms. The total snail count varied among farms (p <0.01) and was 1.80 times higher in the pond for black carp in farm 3 than in the corresponding control pond.

At the end of the nursing cycle, *Bithynia fuchsiana* was recorded from three ponds (8 specimens in total). At post-intervention, counts of thiarid snails varied significantly among farms (p <0.001), while differences between control and intervention ponds was significant only in farm 3; i.e. counts in the pond with black carp was 0.36 times that in the control pond (p <0.001). Viviparid counts differed among farms (p <0.05) and was lower in the ponds with black carp than in the corresponding control ponds i.e. count ratios were 0.87 (not significant), 0.39 (p <0.001) and 0.33 (p <0.001) in farms 1, 2 and 3, respectively. For the total snail counts the pattern was similar to that for the viviparid snails alone, i.e. counts in black carp ponds were 0.89 (n.s.), 0.48 (p <0.001) and 0.36 (p < 0.001) times that in control ponds in farms 1, 2 and 3, respectively.

On average, counts of thiarid snails at pre-intervention was higher in ponds with black carp than in control ponds (count ratio: 1.42, p <0.01) and on average counts in control ponds at termination were 1.63 (p <0.05) times those in control ponds at the beginning, showing that density increased during the nursing cycle. At termination, counts of thiarid snails in black carp ponds were on average 0.65 of those in the control ponds, but that was about the same as in the control ponds at the beginning (count ratio = 0.98, n.s.), showing that density of thiarid snails declined in black carp ponds. Counts of viviparid snails in black carp ponds were on average 0.87 times that in control ponds at the beginning of the experiment. At termination, the counts in control ponds were 2.67 (p < 0.001) times higher than at the beginning, while in black carp ponds, the viviparid counts on average were 0.46 times the counts in control ponds (Figure 1) and that corresponded to 1.24 times the counts in control ponds in the beginning. Thus on average viviparid density increased in both control and black carp ponds during the nursing cycle, but to a lesser extent in black carp ponds than in control ponds..

### Density of snails

**Figure 2:**
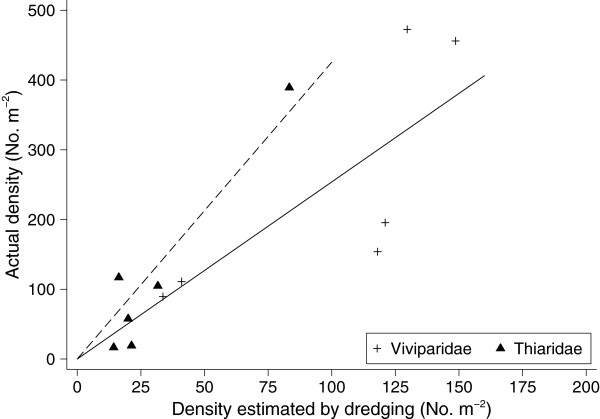
Density of viviparid and thiarid snails in nursery pond in Nam Dinh estimated using 2 different sampling methods.

The quantitative sampling in the emptied pond showed that the dredge sampling underestimated the density (Figure 2). Density of viviparid snails after adjusting for differences among ponds varied significantly with distance from the shore (p <0.001); it was highest close to the shore (0–0.5m) and densities in sections 0.5 - 1.00m, 1.00 - 1.50m and 1.50 - 2.00m respectively were 0.69, 0.37 and 0.24 of that closest to the shore. The same figures for thiarid snails were 0.81, 0.63 and 0.51, respectively.

We attempted to use these densities to calculate a very rough estimate of the standing crop of snails in each pond. Since the pond bottom only sloped slightly beyond 2 m from the shore, average density of samples taken 1.5 - 2.0m from the shore was assumed to be the density for the middle part of the pond (distance >2m from the shore), while the mean of the 6*4 samples was used as density of the section up to 2m from the shore. From several samplings representing the total size variation we have estimated an average wet weight of 0.38g per snail for thiarid snails and 1.5g for viviparid snails. Thus the estimated weight of thiarid snails ranged from 0.8kg to 13.6kg and viviparid snails ranged from 8.6 to 58.8kg in these ponds. Total snail weight ranged from 11.3 to 56.8kg. The estimated maximum consumption by black carp during the entire period would be estimated as average weight (750g/fish) consuming 20% of their body weight per day for 69 days, which would amount to 20.7kg.

### Stomach of black carp content

All stomachs of the black carp were found to be empty. Unfortunately, against prior agreement, farmers decided to remove the juvenile fishes from the nursery ponds about 2 days prior to our final sampling, so we could not collect data to compare juvenile fish production and the seining required to capture the juvenile carps could have disturbed the black carp.

## Discussion

The results show that black carp effectively locate snails and consume large amounts of snails. Thus, in the semi-field experiment, the pond was virtually cleared of snails. The number of snails added in the experimental pond was clearly less than the potential consumption possible by the fishes and snails venturing outside the exclosures would likely be eaten. The effect was less clear in the nursery ponds in Nam Dinh. Although total snail density increased during the experimental time in both control and intervention ponds, the density of thiarid snails declined in the intervention ponds. Stocking density of black carp was high in the experimental pond, while it was probably too low in the nursery ponds to have a more significant impact on snail population density during one nursing cycle (9 weeks). Nico *et al*. [30] reported that small black carp can consume a snail weight equivalent of up to 20% of its body weight per day, but big fish were satiated at about 6%. A rough estimation of the minimum total weight of snails needed for the 15 black carp (700 – 1500g) kept in the experimental would be: 15 fishes x (700 to 1500g) × 6% × 90 days = 56.7 – 121.5kg.

The standing crop of snails in the nursery ponds can be high and could easily exceed the consumption capacity of the two black carp specimens and recruitment to snail populations is also high during the study period. Experiments using black carp to control *Planorbella trivolvis* (Say, 1817) in catfish ponds did not result in significant decline in density as compared to control ponds until 6 months after the introduction of the black carp to the ponds [35]. The farmers in Vietnam normally introduce between 1–5 specimens of black carp per pond depending on pond size (180 – 500 square meters) and density of snails [42]. This stocking density was applied in the nursery ponds of our experiment, while in the semi-field trial, it was 2 and 4 times higher, respectively. Zobel (1975) quoted in [30] gave a formula to calculate the number of black carp required for stocking in different types or sizes of water bodies: M = {[a × (b × 10,000)]/P} × 0.023, where M = stocking density of black carp (fish per hectare), a = the number of molluscs per square meter, b = surface area in hectares, 10,000 = number of square meters per hectare, P = daily consumption of molluscs by black carp, and 0.023 is a constant. However, the value of a and P could be changing with various factors, e.g. season of the year, temperature, prey species composition etc.

In the Red River Delta, black carp is normally used only in grow-out ponds and the purpose is to utilize the snail biomass for fish production [42]; black carp is a valued species for human consumption. Others do it to control snails either because they believe that snails compete with the fishes for food resources or to reduce the nuisance that pointed snail shells constitute when barefooted farmers manage emptied ponds prior to restocking (personal communication with farmers). If snail populations decrease, farmers with black carp may supply snails collected from other habitats as food for the black carp. Thus maintenance of black carp could become a risk factor for infections in fish if the snails collected from small canals or rice fields include FZT intermediate hosts, because infection levels in intermediate host snails in these habitats can be high [26].

In the nursery ponds, the most pronounced reduction in density was observed for the thiarid snails. Hung *et al*. [43] showed that even small black carp can handle fairly large specimens of *M*. *tuberculata*, while larger black carp specimens consumed relatively more viviparid snails and this is probably due to the difference in shell shape, i.e. *M*. *tuberculata* has a much smaller shell diameter than the viviparid snails. Black carp have a relatively small mouth and the size of prey that it can handle depends on its gape size. Shelton *et al*. [44] conducted experiments with various species and sizes of snails by using black carp as a predator and found that fish between 100 and 500 mm (total length) with gape width from 7 to 25 mm could consume snails measuring 7–17 mm in shell diameter. Another reason could be that the shell strength differs among snail species [45,46]. Optimal foraging strategy predicts that molluscivore fishes chose their prey partly depending on a low cost/benefit ratio as measured from crush resistance of the snail shell relative to weight of snail tissue gained [47] and partly on the abundance of snail species [48,49].

The stomachs of the black carp were empty both in the semi field experiment and in the nursery ponds. For the fish from the semi field trial this could be explained by the fact that the fish almost eliminated snails from the pond, while for the nursery ponds farmers had seined all juvenile fishes immediately before termination of experiments and this could have disturbed the black carp. Data reviewed by Nico *et al*. [30] showed that shell fragments are often found in black carp stomachs. During laboratory feeding trials, we, however, observed that the black carp often spat out the crushed snail and then picked up the soft parts thus reducing the amount of shell material ingested. It has been shown that ingestion of shell material is energetically expensive [50].

The implications of our findings is that black carp can control snails in fish ponds, but the standing crop of snails in nursery ponds can be too high for a few black carp specimens to reduce it to a level, where transmission of FZTs is significantly affected during the relatively short nursing cycle (about 9 weeks). It may therefore be necessary to reduce snail densities prior to stocking the nursery ponds with fish fry using pond management, for example through mud and vegetation removal [38]. An alternative would be to increase the stocking density of the black carp considerably. Although overall there is a relationship between infection levels in fishes and either the density of snails or infected snails in nursery ponds, infection levels in fishes can be high even when snail density in ponds is very low [51]. This is probably because nursery ponds often are supplied with water from nearby canals where the density of intermediate hosts and infection levels with FZT in these often is high. It seems that these canals can be important for transmission in ponds depending on water exchange between canals and ponds. Many ponds have a tube connecting it to a canal so that water can go in or out of the pond depending on where water level is highest. The tube has a mesh at both ends preventing passage of fish but cercariae and possibly snails could pass. The tube can be closed by the farmer. Species of the Bithyniidae (especially *Bithynia fuchsiana* and *Parafossarulus manchouricus*), which are intermediate hosts of *Clonorchis sinensis* in this area, can be very abundant especially in small canals [26]. Thus experiments should be conducted to see whether these snails could be controlled in small canals using black carp; this, however, requires close community cooperation because the small canals, support thriving fish populations and are often fished by people (personal observations). The black carp used for snail control should be small specimens, because due to gape limitation they will feed primarily on species of the Thiaridae and Bithynidae, which are important as intermediate hosts for the FZT, but of course they also would feed on juvenile specimens of species of the Viviparidae and Ampullaridae.

Although FZT infections in people can be treated medically [17] the high level of infection in domestic animals like dogs, cats and pigs [52,53] means that transmission persists after treatment of people. Including domestic animals in treatment campaigns would be a major challenge. At least for intestinal FZTs, the short life cycle [54] means that animals should be treated at frequency that is not logistically feasible. Hence farm/pond management [38] including stocking of ponds with black carp could well prove the major intervention to prevent infections in fish and people.

## Conclusions

Black carp can be used for control of snail populations in nursery ponds but pond preparation prior to stocking should reduce snail population density to make it possible for a few black carp specimens to prevent snail populations from building up.

## Competing interests

The authors declared that they have no competing interest.

## Authors’ contributions

NMH, NVD, RJS Jr, HM were involved in development of the study design. NMH, HM performed the experiments. Data analyses were carried out by NMH and HM. All authors read and approved the manuscript.
